# Infantile Fibrosarcoma of the Hand: Limb-Sparing Treatment With Modern Targeted Oral Chemotherapy and Conservative Surgical Resection

**DOI:** 10.1016/j.jhsg.2025.100887

**Published:** 2025-12-05

**Authors:** John R. Vaile, John A. Tipps, Rachel Hurley, Sarah L. Struble, Brooke E. Allen, Lea F. Surrey, Laura S. Finn, Frank M. Balis, Theodore W. Laetsch, Shaun D. Mendenhall

**Affiliations:** ∗ Division of Plastic and Reconstructive Surgery, Department of Surgery, Jefferson Health-Lehigh Valley Hospital, Allentown, PA; † Perelman School of Medicine at the University of Pennsylvania, Philadelphia, PA; ‡ Hematology/Oncology Fellowship Program, Children’s Hospital of Philadelphia, Philadelphia, PA; § Department of Plastic Surgery, University of California, Irvine, Orange, CA; ‖ Division of Plastic and Reconstructive Surgery, Department of Surgery, Spencer Fox Eccles School of Medicine at the University of Utah, and Intermountain Primary Children’s Hospital, Salt Lake City, UT; ¶ Department of Pathology and Laboratory Medicine, Children’s Hospital of Philadelphia, Philadelphia, PA; ∗∗ Division of Oncology, Children’s Hospital of Philadelphia, Philadelphia, PA

**Keywords:** Congenital fibrosarcoma, Infantile fibrosarcoma, Limb salvage, Lorlatinib, Pediatric hand surgery

## Abstract

Infantile fibrosarcoma is a locally aggressive tumor that traditionally requires chemotherapy and radical excision or amputation. Recently, neoadjuvant therapies that exploit its *NTRK* fusion oncogenes have been used to decrease the extent of surgical resection. However, the management of morphologically similar infantile fibrosarcoma-like tumors has not been well characterized. We report a case of an anaplastic lymphoma kinase-driven infantile fibrosarcoma-like neoplasm of the hand that was managed using a multimodal, limb-sparing approach. A 35-week gestation neonate presented with a vascular mass on the volar aspect of his left hand. Neoadjuvant treatment with the anaplastic lymphoma kinase inhibitor lorlatinib led to considerable tumor regression, which enabled conservative surgical resection and preservation of the hand. At 2 years of follow-up, the patient remains on lorlatinib therapy without recurrence and demonstrates excellent hand function despite moderate scar contractures. This case highlights the efficacy of neoadjuvant therapy combined with resection in managing infantile fibrosarcoma-like tumors.

Infantile fibrosarcoma is a rare soft tissue tumor predominantly affecting infants within the first year of life and often necessitates aggressive surgical intervention.^1^, ^2^, ^3^ Historically, chemotherapy and radical surgical excision, including amputation in some cases, has been the primary treatment approach. However, as the majority of infantile fibrosarcomas harbor an *ETV6::NTRK3* fusion oncogene, advancements in neoadjuvant therapies that target the constitutive kinase activation have shown impressive responses, allowing for limb-sparing surgical resection.^3^, ^4^, ^5^ Several reports in the oncology literature support the efficacy of targeted anticancer drugs for infantile fibrosarcoma, with consensus guidelines recommending their use.^6^, ^7^, ^8^, ^9^

Although infantile fibrosarcomas with *NTRK* gene fusions are well documented, there is less information on the management of tumors that have similar morphology (i.e., “infantile fibrosarcoma-like” [IFS-like] neoplasms) but harbor anaplastic lymphoma kinase (ALK) fusions.^9^ The ALK inhibitors are Food and Drug Administration approved for other pediatric mesenchymal tumors with similar genetic signatures. However, their efficacy in ALK-driven infantile fibrosarcoma of the hand has been documented rarely.^10^ To address this gap, we report an ALK-driven IFS-like neoplasm of the hand, which was managed using a multimodal approach with oral lorlatinib, a tyrosine kinase inhibitor that targets ALK, and conservative surgical resection. Written informed consent for this study and images was provided by the participant’s legal guardian.

## Case Report

### Clinical history

**Figure 1:**
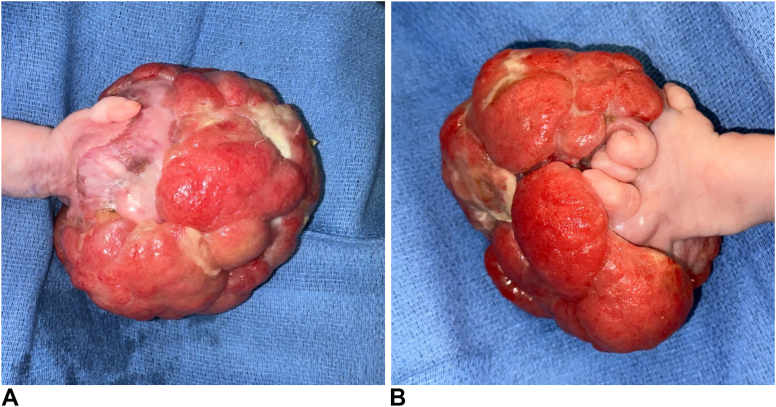
**A** Volar view of infantile fibrosarcoma of the left hand at 7 weeks of age. **B** dorsal view of infantile fibrosarcoma at 7 weeks of age. Printed with permission from and copyrights retained by the principal investigator.

**Figure 2:**
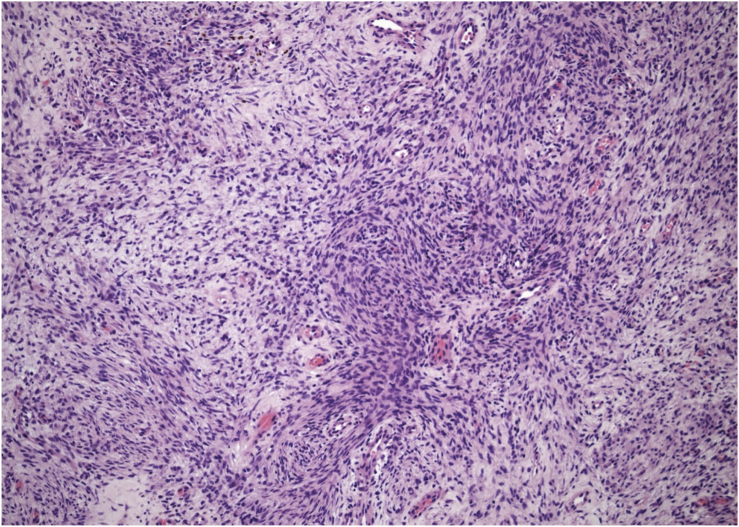
The biopsy showed primitive spindled cells arranged in short interlacing fascicles of variable different cellularity. Extramedullary hematopoiesis and increased vascularity were evident in other regions (Hematoxylin-eosin stain, magnification × 100).

A male infant was born prematurely at 35 weeks’ gestation with a massive vascular mass on the volar aspect of his left hand. After initial treatment at another hospital, he presented to our hospital at 7 weeks of age for further work-up and treatment of the mass (Fig. 1A, B). Ultrasound imaging demonstrated a highly vascular lesion composed of soft tissue and vascular channels concerning for a congenital hemangioma. Magnetic resonance angiography was performed that showed a large, lobulated, and predominantly vascular tumor with multiple tortuous arteries and veins in the hand, forearm, and arm. Tissue biopsy revealed a mitotically active, heterogeneous, moderately vascular, low-grade mesenchymal lesion containing primitive spindled cells of varying density arranged in short fascicles, that lay within a myxoid to hyalinized stroma (Fig. 2). The morphology and immunohistochemical profile, including focal expression of smooth muscle actin and S-100, plus diffuse staining for ALK protein, were compatible with an IFS-like neoplasm. A targeted next generation sequencing cancer fusion panel identified an *EMLA4*::*ALK* gene fusion. Chest computed tomography scan demonstrated an enlarged left axillary lymph node with no pulmonary nodules. Needle biopsy of the enlarged lymph node did not demonstrate metastatic disease.

### Medical treatment

**Figure 3:**
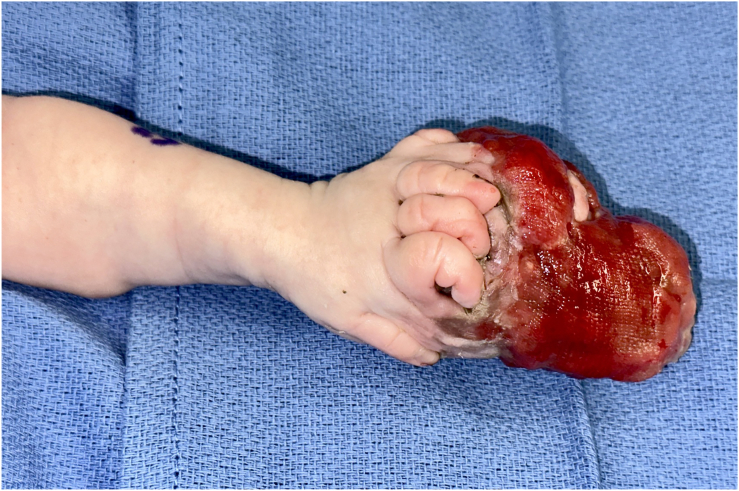
Dorsal view of infantile fibrosarcoma at the time of initial resection, following 15 days of lorlatinib therapy. Printed with permission from and copyrights retained by the principal investigator.

**Figure 4:**
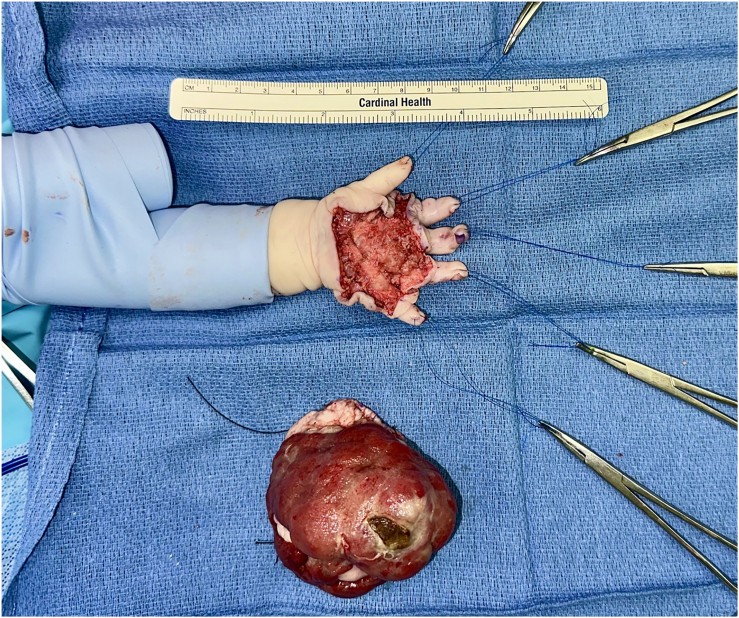
Volar view of palmar defect following mass resection and tumor specimen. Printed with permission from and copyrights retained by the principal investigator.

**Figure 5:**
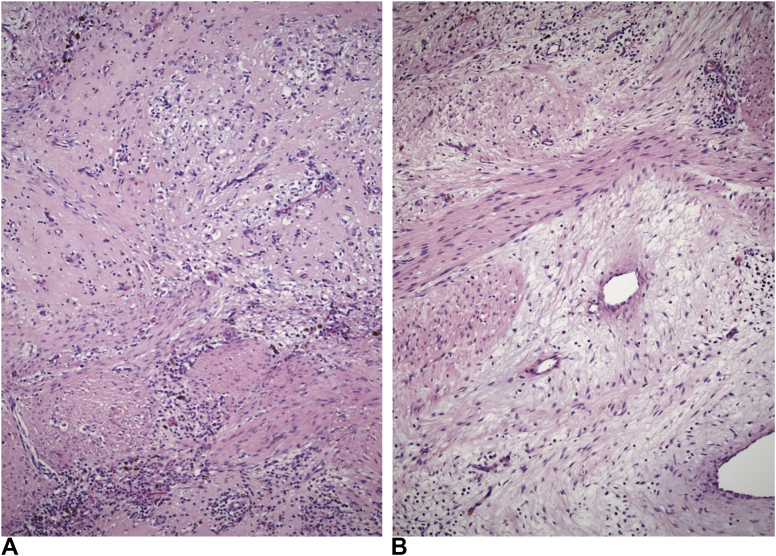
After treatment with lorlatinib, many regions of the resected neoplasm were less cellular, contained more abundant glassy hyalinized **A** or edematous **B** stroma and showed apparent “maturation” of spindled cells such that they resembled fibroblasts. (Hematoxylin-eosin stain, magnification × 100 and magnification × 200).

Before surgery, the patient was started on the oral ALK inhibitor lorlatinib at a dose of 3.83 mg/kg daily. The goal of therapy was to shrink the tumor to facilitate surgical resection without sacrificing critical hand structures such as the digital neurovascular bundles and tendons. There was notable tumor response after 15 days of lorlatinib (Fig. 3), at which time he underwent surgical resection of the mass (Fig. 4). Lorlatinib was only held on the day of the surgery. Pathology of the tumor from resection showed decreased cellularity and stromal consolidation consistent with effective targeted therapy (Fig. 5A, B).

### Surgical treatment

The initial surgery was performed at 3 months of age. Most of the tumor was resected, preserving all the neurovascular bundles and tendons, and a skin substitute was used to cover the cutaneous defect of the palm while awaiting margins from permanent pathology. Deep margins were positive for residual tumor. Two weeks later, the patient returned to the operating room for additional conservative tumor debulking at the deep margin and full-thickness skin grafting. The sample contained scar and markedly inflamed granulation tissue, rare spindled cells, and occasional small vessels that demonstrated ALK expression. Because of the positive margins, a multidisciplinary decision was made to continue with lorlatinib therapy because of its efficacy and not to pursue further resection of the tumor along with critical structures of the hand.

### Follow-up

At the 2-week follow-up visit, the patient had loss of approximately 50% of the full-thickness skin graft and the resulting wound was allowed to heal secondarily. This resulted in a 90° flexion contracture deformity of the ring finger proximal interphalangeal joint and a 30° contracture of the small finger proximal interphalangeal joint. There were no other complications in the perioperative period. Three months after initial skin grafting, the patient returned to the operating room for scar contracture release, percutaneous pinning of the ring and small fingers, and full-thickness skin grafting. At 19 months of age, he returned to the operating room for additional scar contracture release and full-thickness grafting. After each scar release surgery, hand therapy was recommended to maximize range of motion and function.

### Outcome

**Figure 6:**
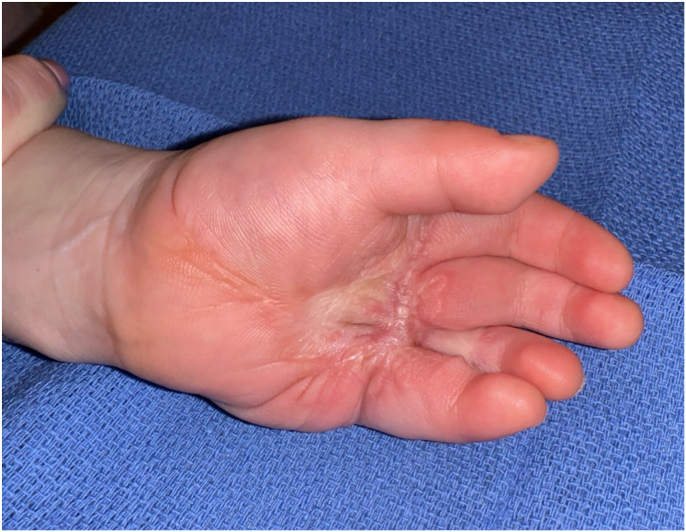
Post-treatment, volar view at 2-year follow-up visit. Printed with permission from and copyrights retained by the principal investigator.

**Figure 7:**
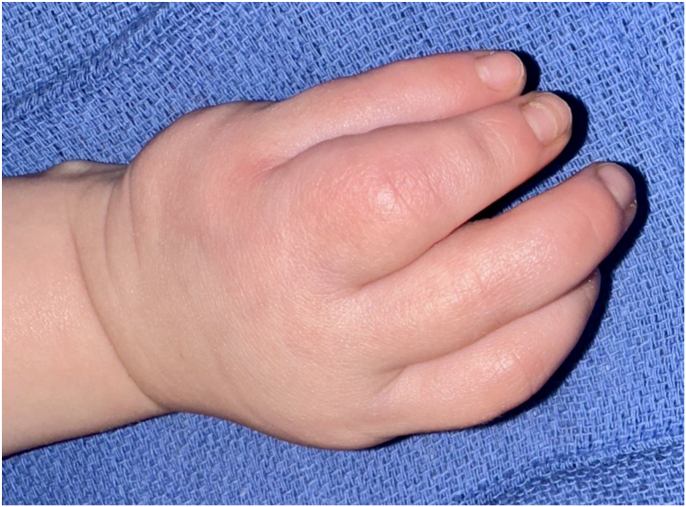
Post-treatment, dorsal view at 2-year follow-up visit. Printed with permission from and copyrights retained by the principal investigator.

At 2-years of follow-up, the patient continues daily lorlatinib therapy with dose adjustments for weight. Lorlatinib therapy has been well tolerated with a stable elevation in cholesterol and triglycerides that have not required intervention. The patient has shown no evidence of recurrent or metastatic disease on recent imaging, and he has a strong grasp with nearly equivalent use of his left and right hands. A moderate scar contracture recurred transversely between the middle and small fingers, with mild flexion contracture (Figure 6, Figure 7). Integrating lorlatinib therapy with staged surgical interventions led to a remarkable aesthetic and functional outcome as well as avoided hand amputation.

## Discussion

Infantile fibrosarcoma and IFS-like neoplasms of the hand present a formidable management challenge because of locally aggressive behavior and the potential need for amputation.^3^^,^^4^^,^^10^ Traditionally, the management of these tumors depended on radical surgical resection, which can result in considerable morbidity, including partial or full limb loss.^2^^,^^3^^,^^10^ The use of targeted kinase inhibitors has changed this treatment paradigm.^3^^,^^4^^,^^6^, ^7^, ^8^, ^9^ In a phase 1 trial of larotrectinib, a selective tropomycin receptor kinase (TRK) inhibitor, in patients with relapsed or nonresponsive solid tumors or central nervous system tumors, or infantile fibrosarcoma that would otherwise require disfiguring surgery, 14 of 15 patients (93%; 95% CI, 68–100) patients with a TRK fusion had an objective response.^7^ Eight of these patients had infantile fibrosarcoma. Entrectinib (a TRK, ROS1, and ALK inhibitor,) in a phase 1/2 trial of children with extracranial solid or central nervous system tumors demonstrated an objective response rate of 60% (9 of 15 patients; 95% CI, 32.3–83.7%) in patients with *NTRK1/2/3* fusions and 33% (1 of 3 patients; 95% CI, 0.84–90.6%) in patients with *ALK* fusions.^7^

Duration of therapy remains an ongoing area of investigation broadly for targeted kinase inhibitors. Within the phase 1 trial of larotrectinib, 5 patients with locally advanced soft tissue sarcoma (2) or infantile fibrosarcoma (3) received neoadjuvant larotrectinib with partial response before surgical resection. Of the patients with infantile fibrosarcoma, 2 had a complete resection with clear margins and did not continue therapy after surgery, remaining disease-free for 15 and 12 months respectively. The third patient had an R1 resection with viable tumor at the margins, prompting resumption of larotrectinib.^5^ In our case, positive margins were left to optimize hand function, prompting continuation of lorlatinib therapy.

The ability to shrink these tumors dramatically before surgery with inhibitors of the driving kinase fusion substantially reduces the risk of surgical morbidity.^4^^,^^5^ In this patient’s case, lorlatinib therapy resulted in considerable tumor reduction, thereby allowing for staged surgical resection with preservation of the hand with an acceptable aesthetic and functional outcome. The staged approach involved multiple debulking procedures, placement of a skin substitute, and subsequent full-thickness skin grafting. This combination yielded complete response at 2-year follow-up with only moderate flexion and transverse scar contractures. The early success of this approach highlights the importance of a multidisciplinary team comprising oncology, hand surgery, radiology, pathology, and hand therapy working collaboratively to optimize hand function and quality of life. This approach aligns with other recent literature emphasizing the benefits of preoperative treatment in facilitating limb-sparing surgical resection across different types of pediatric soft tissue sarcomas.^3^^,^^4^

This study has several limitations. Despite favorable results at 2-year follow-up, there is a need for continued surveillance to assess the durability of this treatment and its impact on long-term functional outcomes. The optimal duration of lorlatinib therapy is not defined in this situation. Given limited side effects in this patient, they continue on lorlatinib at this time. It is anticipated that the child will require additional scar contracture releases and hand therapy as he grows. Furthermore, longer term follow-up is necessary to monitor tumor recurrence, especially when therapy is withdrawn, late effects of systemic therapy, and overall trends in global hand function.

Multidisciplinary management of this case of infantile fibrosarcoma of the hand, combining neoadjuvant modern targeted oral chemotherapy with staged conservative surgical resection, led to successful tumor eradication and near full hand function at 2-year follow-up.

## Conflicts of Interest

Dr Mendenhall is an educational consultant for PolyNovo. No benefits in any form have been received or will be received by the other authors related directly to this article.
